# Age modifies recurrence risk across the 2025 American Thyroid Association risk stratification in papillary thyroid carcinoma

**DOI:** 10.1210/jendso/bvag259

**Published:** 2026-11-12

**Authors:** Li’nan Qin, Yunhe Liu, Xiwei Zhang, Dangui Yan, Jie Liu, Wensheng Liu, Song Ni, Hui Huang, Shaoyan Liu

**Affiliations:** Department of Head and Neck Surgical Oncology, National Cancer Centre/National Clinical Research Centre for Cancer/Cancer Hospital, Chinese Academy of Medical Sciences and Peking Union Medical College, Beijing 100021, China; Department of Head and Neck Surgical Oncology, National Cancer Centre/National Clinical Research Centre for Cancer/Cancer Hospital, Chinese Academy of Medical Sciences and Peking Union Medical College, Beijing 100021, China; Department of Head and Neck Surgical Oncology, National Cancer Centre/National Clinical Research Centre for Cancer/Cancer Hospital, Chinese Academy of Medical Sciences and Peking Union Medical College, Beijing 100021, China; Department of Head and Neck Surgical Oncology, National Cancer Centre/National Clinical Research Centre for Cancer/Cancer Hospital, Chinese Academy of Medical Sciences and Peking Union Medical College, Beijing 100021, China; Department of Head and Neck Surgical Oncology, National Cancer Centre/National Clinical Research Centre for Cancer/Cancer Hospital, Chinese Academy of Medical Sciences and Peking Union Medical College, Beijing 100021, China; Department of Head and Neck Surgical Oncology, National Cancer Centre/National Clinical Research Centre for Cancer/Cancer Hospital, Chinese Academy of Medical Sciences and Peking Union Medical College, Beijing 100021, China; Department of Head and Neck Surgical Oncology, National Cancer Centre/National Clinical Research Centre for Cancer/Cancer Hospital, Chinese Academy of Medical Sciences and Peking Union Medical College, Beijing 100021, China; Department of Head and Neck Surgical Oncology, National Cancer Centre/National Clinical Research Centre for Cancer/Cancer Hospital, Chinese Academy of Medical Sciences and Peking Union Medical College, Beijing 100021, China; Department of Head and Neck Surgical Oncology, National Cancer Centre/National Clinical Research Centre for Cancer/Cancer Hospital, Chinese Academy of Medical Sciences and Peking Union Medical College, Beijing 100021, China

**Keywords:** papillary thyroid cancer, recurrence, American thyroid association, age, risk stratification

## Abstract

**Context:**

The 2025 American Thyroid Association (ATA) guidelines updated recurrence risk stratification for papillary thyroid cancer (PTC). Whether age modifies the absolute structural recurrence risk represented by these strata is unclear.

**Objective:**

To determine whether age modifies 5-year structural recurrence risk across ATA 2025 stratification in PTC and improves risk prediction.

**Methods:**

This retrospective cohort study included 14 397 patients with PTC who underwent initial surgery at a tertiary cancer center in China from 2000 to 2018. The primary outcome was 5-year structural recurrence-free survival. Cox models with restricted cubic splines evaluated age effects and ATA2025-by-age interaction. ATA-based and age-interaction models were compared by temporal validation and decision-curve analysis.

**Results:**

The median age was 43.0 years, 10 867 patients (75.5%) were female, and 758 (5.3%) experienced structural recurrence during a median follow-up of 60.0 months. Observed 5-year risk rose from 1.7% to 12.5% across ATA2025 stratification. Age modified risk across stratifications (global interaction *P* < .001). After treatment adjustment, 5-year risk fell from 2.37% at age 35 years to 0.69% at age 65 years in the low-risk group but rose from 8.21% to 19.28% in the high-risk group. In temporal validation, the C-index was 0.745 for both models; 5-year areas under the curve were 0.743 and 0.747, and Brier scores were 0.043 and 0.042. Decision curves showed little overall difference.

**Conclusion:**

Age identified meaningful heterogeneity in absolute structural recurrence risk, particularly in high-risk PTC, but added little to predictive performance or decision value. External validation is required before age is used to guide treatment or surveillance.

Thyroid cancer is the most common malignancy of the endocrine system worldwide. In 2022, ∼821 000 new cases were estimated worldwide, corresponding to an age-standardized incidence rate of 9.1 per 100 000 population; China accounted for ∼466 100 cases, making thyroid cancer the third most commonly diagnosed cancer nationally (1-3). Most thyroid cancers are differentiated thyroid carcinomas (DTC), particularly papillary thyroid cancers (PTC) (3). Although most patients with PTC have excellent disease-specific survival after initial treatment, structural recurrence remains clinically important because it leads to repeated imaging, additional surgery, radioactive iodine treatment, long-term surveillance, and persistent patient anxiety (3, 4). Therefore, accurate estimation of structural recurrence risk is central to postoperative counseling and follow-up planning.

The American Thyroid Association (ATA) recurrence risk stratification system is widely used to estimate the likelihood of persistent or recurrent structural disease after initial therapy (4, 5). The 2025 ATA guidelines refined postoperative recurrence risk assessment for adult patients with DTC by updating the recurrence risk stratification and related clinicopathologic criteria (4). However, this stratification is often interpreted as representing fixed levels of recurrence risk, although the absolute recurrence risk assigned to a given stratum may differ across patient characteristics.

Age is an established prognostic factor in PTC mortality staging, but its role in postoperative structural recurrence risk remains less clearly defined (6-13). Some studies suggest that age is associated with recurrence, particularly among patients with intermediate- or high-risk disease, but whether age modifies the absolute recurrence risk represented by the updated ATA2025 strata has not been defined.

The primary objective was to estimate age-specific 5-year structural recurrence risks within each ATA 2025 stratification. We further examined whether the association persisted after accounting for treatment and surgical period and whether an age-interaction model improved temporal-validation performance, decision-curve net benefit, and clinically interpretable risk reclassification. This design separates evidence of biological or prognostic heterogeneity from evidence that age should be incorporated into routine clinical risk prediction.

## Materials and methods

### Study design and population

This retrospective cohort study reviewed the medical records of patients diagnosed with PTC who underwent initial surgery at the National Cancer Center/Cancer Hospital, Chinese Academy of Medical Sciences and Peking Union Medical College, between January 2000 and December 2018. Among 18 785 patients evaluated, 14 397 had complete clinicopathological variables required for 2025 ATA recurrence-risk stratification system (ATA 2025) and complete follow-up data. These patients constituted the analytical cohort.

Patients were excluded if they had incomplete clinicopathological or follow-up data, underwent noncurative or palliative surgery, had pT4b disease, an R2 resection or distant metastasis at presentation, or were followed for <12 months. Thus, the analysis was restricted to patients treated with curative intent and without known unresected locoregional or distant disease at initial treatment. Missing pathological variables were not imputed.

The study followed the Strengthening the Reporting of Observational Studies in Epidemiology guidelines and was conducted in accordance with the Declaration of Helsinki. This was a retrospective analysis of routinely collected medical records and involved no study-specific intervention. Ethical oversight applied to the access, deidentification, analysis, and reporting of data from patients treated between 2000 and 2018. The protocol for this retrospective analysis was approved by the Ethics Committee of the National Cancer Center/Cancer Hospital, Chinese Academy of Medical Sciences and Peking Union Medical College on July 8, 2025 (approval no. 25/328-5274), and all analytical data were deidentified before analysis.

### ATA 2025 risk assignment and treatment variables

The primary exposure was ATA2025 recurrence risk stratification, classified as low, low-intermediate, intermediate-high, or high risk. ATA2025 stratification was retrospectively assigned using available pathological and clinical variables, including tumor size, aggressive histologic variant, lymphovascular invasion (LVI), extrathyroidal extension (ETE), extranodal extension (ENE), multifocality, metastatic lymph node burden, lateral lymph node metastasis, and pathological T and N stage.

All algorithm-generated assignments were subsequently reviewed by 2 thyroid surgeons against the available source records. Only patients with the complete set of variables required for ATA 2025 stratification were included, and no missing pathological variable was imputed or assumed to be absent.

Treatment variables included the extent of thyroid surgery (lobectomy or total thyroidectomy), central neck dissection (none, unilateral, or bilateral), and postoperative treatment (thyroid-stimulating hormone suppression alone, radioactive iodine or external-beam radiotherapy). Radioactive iodine and external-beam radiotherapy were recorded and analyzed separately. Because ATA 2025 stratification was assigned retrospectively, it did not guide the initial treatment decisions.

### Surveillance and outcome ascertainment

Patients were scheduled for physical examination and neck ultrasonography every 6 months during the first 5 postoperative years and annually thereafter. Chest computed tomography or radiography was performed annually. Structural recurrence in the remaining thyroid or thyroid bed, or in the central or lateral cervical lymph nodes, was confirmed by cytology or histopathology. Distant metastasis was diagnosed primarily by computed tomography or emission computed tomography. Brain magnetic resonance imaging or bone scanning was performed when clinically indicated.

The study focused on structural recurrence. Thyroglobulin and antithyroglobulin antibody levels were not used as diagnostic criteria for recurrence, and biochemical abnormalities without identifiable structural disease were not classified as events.

Follow-up information was obtained primarily from electronic medical records. Telephone contact was used to supplement incomplete follow-up records and to ascertain vital status when death information was incomplete.

The primary endpoint was structural recurrence-free survival (RFS), defined as the interval from initial surgery to the first confirmed structural recurrence. Patients who died without documented recurrence were censored at the date of death, and patients who remained recurrence-free were censored at their last follow-up. The primary prediction horizon was 60 months.

### Statistical analysis

Baseline characteristics were summarized overall and by ATA2025 stratification. Continuous variables were reported as median with interquartile ranges, and categorical variables as counts and percentages. Kaplan–Meier analysis was used to estimate RFS and 5-year structural recurrence risk. Cox proportional hazards regression was used to estimate hazard ratios and 95% confidence intervals for ATA2025 stratification, with the low-risk group as reference.

The primary analysis evaluated whether age modified structural recurrence risk within the ATA2025 stratification. Age was modeled as a continuous variable using restricted cubic splines with 3 knots. Knots were placed at the 10th, 50th, and 90th percentiles of the age distribution, corresponding to ages 29, 43, and 58 years.

Three nested Cox models were fitted. The ATA-based model included ATA2025 stratification, sex, and surgery-year period. The age model additionally included spline-modeled age. The age-interaction model further included interactions between ATA2025 stratification and spline-modeled age. Surgical period was categorized as 2000-2009, 2010-2014, or 2015-2018. Nested models were compared using likelihood-ratio tests, with the comparison between models with and without the interaction terms providing the global test of effect modification. Tied event times were handled using the Efron method. The proportional-hazards assumption was evaluated using Schoenfeld residuals.

Age-specific predicted 5-year structural recurrence risks were estimated at prespecified ages of 35, 45, 55, and 65 years. Estimates from the primary model were marginally standardized over the empirical joint distribution of sex and surgical period. Within each ATA2025 stratum, the risk difference was calculated as the predicted 5-year risk at age 65 years minus that at age 35 years. Confidence intervals for standardized risks and risk differences were derived from 2000 coefficient simulations based on the estimated variance–covariance matrix.

A treatment-adjusted model additionally included surgery extent, central neck dissection and postoperative treatment, with radioactive iodine and external-beam radiotherapy represented separately. Estimates from this model were standardized over the observed distributions of these treatment variables.

Variation across surgical periods was evaluated using a hierarchical ATA 2025-by-age-by-period interaction model that retained all lower-order terms. Models with and without the three-way interaction were compared using a likelihood-ratio test. Treatment-adjusted age effects were also estimated separately within each surgical period.

### Temporal validation, reclassification, and decision-curve analysis

Temporal validation was performed by fitting the models among patients treated from 2000 to 2016 and evaluating their performance among patients treated from 2017 to 2018. Restricted cubic spline knots were estimated in the derivation cohort and retained without modification when predictions were generated for the validation cohort.

Model performance was assessed using Harrell C-index, the inverse-probability-of-censoring-weighted 5-year time-dependent area under the receiver operating characteristic curve, the 5-year Brier score, and the calibration slope. Agreement between mean predicted and Kaplan–Meier-estimated 5-year risks was also assessed. Calibration was further examined across quantile groups of predicted risk. Confidence intervals for validation metrics were obtained from 300 bootstrap resamples of the validation cohort.

Decision-curve analysis compared the ATA-based and age-interaction models in the temporal validation cohort. Net benefit was evaluated at 5 years across threshold probabilities from 1% to 20% in increments of 0.5 percentage points. Inverse-probability-of-censoring weighting was used to account for censoring.

Risk reclassification was assessed using prespecified 5-year risk categories of <2%, 2% to <5%, 5% to <10%, and 10% or greater. Movement to a lower, unchanged or higher category was summarized overall and within each original ATA 2025 stratum. Full-cohort reclassification was considered an apparent within-cohort analysis. In the temporal validation cohort, observed 5-year risks within the reclassification groups were estimated using Kaplan–Meier methods.

### Sensitivity analyses

Sensitivity analyses evaluated restricted cubic splines with 4 knots, a linear age term, and age cutoffs of 45 and 55 years. Additional analyses included adjustment for treatment variables; restriction to patients with at least 24 months of follow-up or recurrence within the first 24 months; restriction to patients receiving thyrotropin suppression alone; and exclusion of patients older than 70 or 75 years. A further analysis substituted the recorded last follow-up time for censored observations whose analysis time differed from their last follow-up time.

All analyses were performed using R version 4.4.2, principally with the survival package version 3.8-6 and Hmisc package version 5.2-5. All statistical tests were two-sided, and (*P* < .05) was considered statistically significant.

## Results

### Cohort characteristics and treatment

Among 18 785 patients evaluated, 4388 were excluded because of incomplete clinicopathological or follow-up data or other prespecified exclusion criteria (Fig. S1) (14). The final cohort included 14 397 patients with a median age of 43.0 years (IQR, 35.0-51.0), of whom 75.5% were female. The median tumor size was 0.9 cm (IQR: 0.6-1.5). Unilateral and bilateral multifocal PTC were observed in 14.4% (*n* = 2076) and 26.2% (*n* = 3773) of patients, respectively. LVI was present in 1.7% (*n* = 251) and HT in 22.9% (*n* = 3291) of patients. Microscopic and gross ETE were present in 40.2% and 10.3% of patients, respectively, while ENE was noted in 12.4%. Aggressive histological variants were rare (0.9%). Pathological T stages were predominantly pT1a (58.5%) and pT1b (24.2%), followed by pT2 (6.4%), pT3b (5.8%), pT4a (4.4%), and pT3a (0.6%). The median number of metastatic lymph node number was 1.0 (IQR: 0-4.0) (Table 1). The cohort comprised 4126 patients (28.7%) in the ATA 2025 low-risk group, 4765 (33.1%) in the low-intermediate-risk group, 2649 (18.4%) in the intermediate-high-risk group and 2857 (19.8%) in the high-risk group. Clinicopathological characteristics according to age group and surgical period are presented in Tables S1 and S2 (14).

**Table 1:** Baseline characteristics of the study cohort according to ATA2025 recurrence risk stratification

Characteristic	Overall (*n* = 14 397)	Low (*n* = 4126)	Low-intermediate (*n* = 4765)	Intermediate-high (*n* = 2649)	High (*n* = 2857)
Age, median (IQR), y	43.0 (35.0-51.0)	43.0 (35.0-51.0)	45.0 (37.0-52.0)	41.0 (32.0-50.0)	43.0 (34.0-53.0)
Female sex	10 867 (75.5)	3206 (77.7)	3725 (78.2)	1946 (73.5)	1990 (69.7)
Tumor size, median (IQR), cm	0.9 (0.6-1.5)	0.6 (0.5-1.0)	0.8 (0.5-1.0)	1.2 (0.8-1.8)	1.5 (1.0-2.5)
Metastatic lymph nodes, median (IQR)	1.0 (0.0-4.0)	0.0 (0.0-1.0)	0.0 (0.0-2.0)	4.0 (1.0-9.0)	6.0 (2.0-14.0)
Aggressive histologic variant	135 (0.9)	0	0	53 (2.0)	82 (2.9)
Lymphovascular invasion	251 (1.7)	0	2 (<0.1)	118 (4.5)	131 (4.6)
Hashimoto thyroiditis	3291 (22.9)	939 (22.8)	1162 (24.4)	754 (28.5)	436 (15.3)
Microscopic extrathyroidal extension	5791 (40.2)	0 (0.0)	3083 (64.7)	1716 (64.8)	992 (34.7)
Gross extrathyroidal extension	1478 (10.3)	0	0	0	1478 (51.7)
Extranodal extension	1791 (12.4)	0	0	0	1791 (62.7)
Unifocal disease	8548 (59.4)	4126 (100.0)	2411 (50.6)	727 (27.4)	1284 (44.9)
Unilateral multifocal	2076 (14.4)	0 (0.0)	905 (19.0)	769 (29.0)	402 (14.1)
Bilateral multifocal	3773 (26.2)	0 (0.0)	1449 (30.4)	1153 (43.5)	1171 (41.0)
pT1 stage	11 909 (82.7)	3949 (95.7)	4584 (96.2)	2263 (85.4)	1113 (38.9)
pT2 stage	923 (6.4)	177 (4.3)	163 (3.4)	371 (14.0)	212 (7.4)
pT3 stage	928 (6.4)	0 (0.0)	18 (0.4)	15 (0.6)	895 (31.3)
pT4a stage	637 (4.4)	0 (0.0)	0 (0.0)	0 (0.0)	637 (22.3)
pN1a stage	4405 (30.6)	1040 (25.2)	1774 (37.2)	793 (29.9)	798 (27.9)
pN1b stage	3281 (22.8)	92 (2.2)	268 (5.6)	1254 (47.3)	1667 (58.3)
Lobectomy	8244 (57.3)	3568 (86.5)	2637 (55.3)	1013 (38.2)	1026 (35.9)
Central neck dissection	12 559 (87.2)	3012 (75.1)	4286 (89.9)	2510 (94.8)	2661 (93.1)
RAI	931 (6.5)	7 (0.2)	88 (1.8)	221 (8.3)	615 (21.5)
External-beam radiotherapy	10 (0.1)	0 (0.0)	0 (0.0)	0 (0.0)	10 (0.4)
Follow-up, median (IQR), months	60.0 (29.0-86.0)	60.0 (35.0-90.0)	57.0 (25.0-81.0)	60.0 (28.0-85.0)	60.0 (35.0-94.0)
Structural recurrence	758 (5.3)	89 (2.2)	120 (2.5)	143 (5.4)	406 (14.2)

Values are presented as *n* (%) unless otherwise indicated; continuous variables are presented as median (interquartile range). ATA, American Thyroid Association; IQR, interquartile range; pN, pathological nodal stage; pT, pathological tumor stage; RAI, radioactive iodine.

Lobectomy was performed in 8244 patients (57.3%), including 1026 of 2857 high-risk patients (35.9%). Central neck dissection was performed in 12 559 patients (87.2%). Radioactive iodine was administered to 931 patients (6.5%), including 615 high-risk patients (21.5%). External-beam radiotherapy was administered to 10 patients, all in the high-risk group (Fig. S2 and Table S3) (14).

### ATA 2025 recurrence risk and modification by age

Over a median follow-up of 60.0 months (IQR, 29.0-86.0), structural recurrence occurred in 758 patients (5.3%). The overall RFS was 97.1% at 3 years and 95.2% at 5 years. Kaplan–Meier analyses demonstrated progressive separation of structural RFS across ATA2025 recurrence risk stratification (Fig. 1). The estimated 5-year RFS was 98.3% (95% CI 97.9-98.7) in the low-risk group, 97.4% (95% CI 96.7-97.8) in the low-intermediate-risk group, 95.0% (95% CI 93.9-95.9) in the intermediate-high-risk group, and 87.5% (95% CI 86.1-88.8) in the high-risk group. Corresponding 5-year structural recurrence risks were 1.7% (95% CI 1.3-2.1), 2.6% (95% CI 2.2-3.3), 5.0% (95% CI 4.1-6.1), and 12.5% (95% CI 11.2-13.9), respectively.

**Figure 1:**
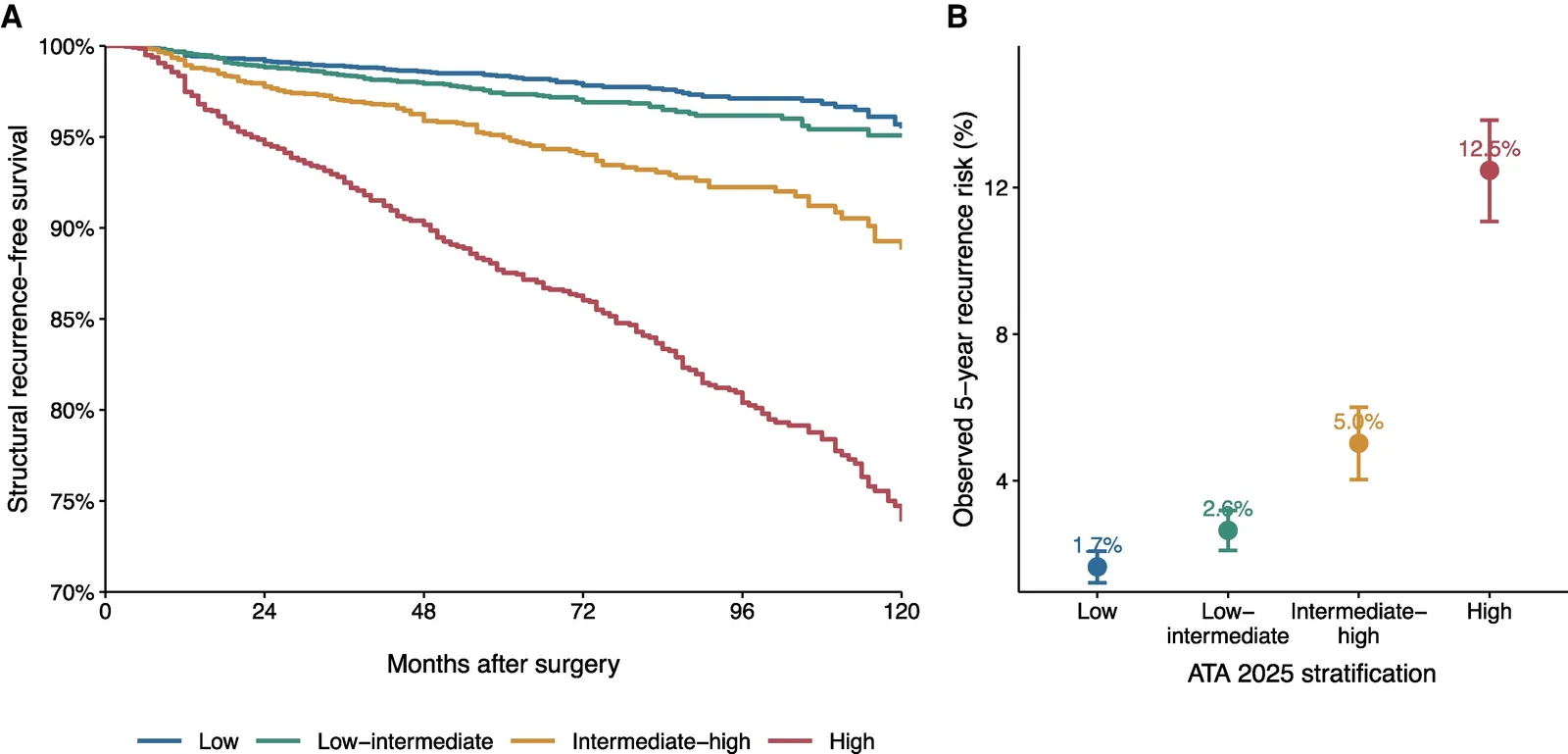
Observed performance of ATA2025 recurrence risk stratification. (A) Kaplan–Meier estimates of structural RFS according to ATA 2025 stratification during the first 120 months after surgery. (B) Observed 5-year structural recurrence risks with 95% confidence intervals. Abbreviations: ATA, American Thyroid Association; CI, confidence interval; RFS, recurrence-free survival.

Using the low-risk group as the reference, the HR for structural recurrence was 1.42 (95% CI 1.08-1.87; *P* = .012) for low-intermediate-risk, 2.87 (95% CI 2.20-3.74; *P* < .001) for intermediate-high risk, and 7.09 (95% CI 5.64-8.92; *P* < .001) for high risk. The global test for ATA2025 stratification was significant (*P* < .001).

Age modified the association between ATA 2025 stratification and recurrence risk (global interaction *P* < .001). In the primary model, the standardized 5-year risk in the low-risk group decreased from 2.38% (95% CI 1.88-3.01) at age 35 years to 0.68% (0.28-1.54) at age 65 years, corresponding to a risk difference of −1.70 percentage points (95% CI −2.59 to −0.53) and a hazard ratio of 0.28 (0.10-0.77). The corresponding risk difference was −0.87 percentage points (−2.19 to 0.98) in the low-intermediate-risk group and 0.23 percentage points (−2.56 to 4.22) in the intermediate-high-risk group. In the high-risk group, standardized risk increased from 9.06% (7.85-10.40) at age 35 years to 20.08% (17.08-23.91) at age 65 years, a difference of 11.02 percentage points (7.79-14.84; hazard ratio, 2.37 [1.90-2.96]) (Table 2 and Fig. S3) (14).

**Table 2:** Standardized 5-year structural recurrence risk at prespecified ages

Model	ATA 2025 risk	Age 35 years, % (95% CI)	Age 45 years, % (95% CI)	Age 55 years, % (95% CI)	Age 65 years, % (95% CI)	Risk difference: age 65 minus 35 years, pp (95% CI)
Primary model	Low	2.38 (1.88-3.01)	1.61 (1.22-2.06)	1.05 (0.72-1.52)	0.68 (0.28-1.54)	−1.70 (−2.59 to −0.53)
Primary model	Low-intermediate	3.10 (2.57-3.75)	2.12 (1.71-2.67)	2.05 (1.60-2.66)	2.23 (1.28-3.84)	−0.87 (−2.19 to +0.98)
Primary model	Intermediate-high	4.92 (4.02-6.05)	3.93 (3.08-4.97)	4.28 (3.24-5.64)	5.15 (2.90-8.71)	+0.23 (−2.56 to +4.22)
Primary model	High	9.06 (7.85-10.40)	8.59 (7.33-10.06)	12.39 (10.86-14.16)	20.08 (17.08-23.91)	+11.02 (+7.79 to +14.84)
Treatment-adjusted model	Low	2.37 (1.92-2.97)	1.62 (1.24-2.13)	1.06 (0.73-1.59)	0.69 (0.30-1.64)	−1.68 (−2.55 to −0.48)
Treatment-adjusted model	Low-intermediate	3.20 (2.62-3.93)	2.22 (1.79-2.76)	2.17 (1.69-2.77)	2.36 (1.36-4.03)	−0.84 (−2.34 to +1.06)
Treatment-adjusted model	Intermediate-high	4.86 (4.04-5.92)	3.98 (3.18-5.05)	4.35 (3.29-5.78)	5.19 (3.05-9.19)	+0.33 (−2.46 to +4.54)
Treatment-adjusted model	High	8.21 (7.14-9.54)	7.97 (6.87-9.40)	11.71 (10.23-13.62)	19.28 (16.27-23.02)	+11.07 (+7.98 to +14.55)

The primary model included spline-modeled age, ATA 2025 stratification, their interaction, sex, and surgical period. The treatment-adjusted model additionally included the extent of thyroid surgery, central neck dissection, and postoperative treatment, with radioactive iodine and external-beam radiotherapy modeled separately. Estimates were standardized over the empirical covariate distribution. Risks and 95% confidence intervals were derived from 2000 coefficient simulations. Risk differences compare the estimated risk at age 65 years with that at age 35 years and are expressed in percentage points. ATA, American Thyroid Association; CI, confidence interval; pp, percentage points.

### Treatment adjustment, surgical periods, and sensitivity analyses

Adjustment for surgery extent, central neck dissection and postoperative treatment produced similar age-specific estimates (global interaction *P* < .001). In the low-risk group, treatment-adjusted 5-year risk decreased from 2.37% (95% CI 1.92-2.97) at age 35 years to 0.69% (0.30-1.64) at age 65 years (risk difference, −1.68 percentage points [−2.55 to −0.48]). In the high-risk group, risk increased from 8.21% (7.14-9.54) to 19.28% (16.27-23.02), a difference of 11.07 percentage points (7.98-14.55; hazard ratio, 2.52 [2.01-3.15]) (Fig. 2 and Table 2). Detailed primary and treatment-adjusted age effects and the associations of individual treatment covariates with structural recurrence are reported in Tables S4A and S4B (14). The global Schoenfeld-residual test did not indicate nonproportional hazards (*P* = .178; Table S4C) (14).

**Figure 2:**
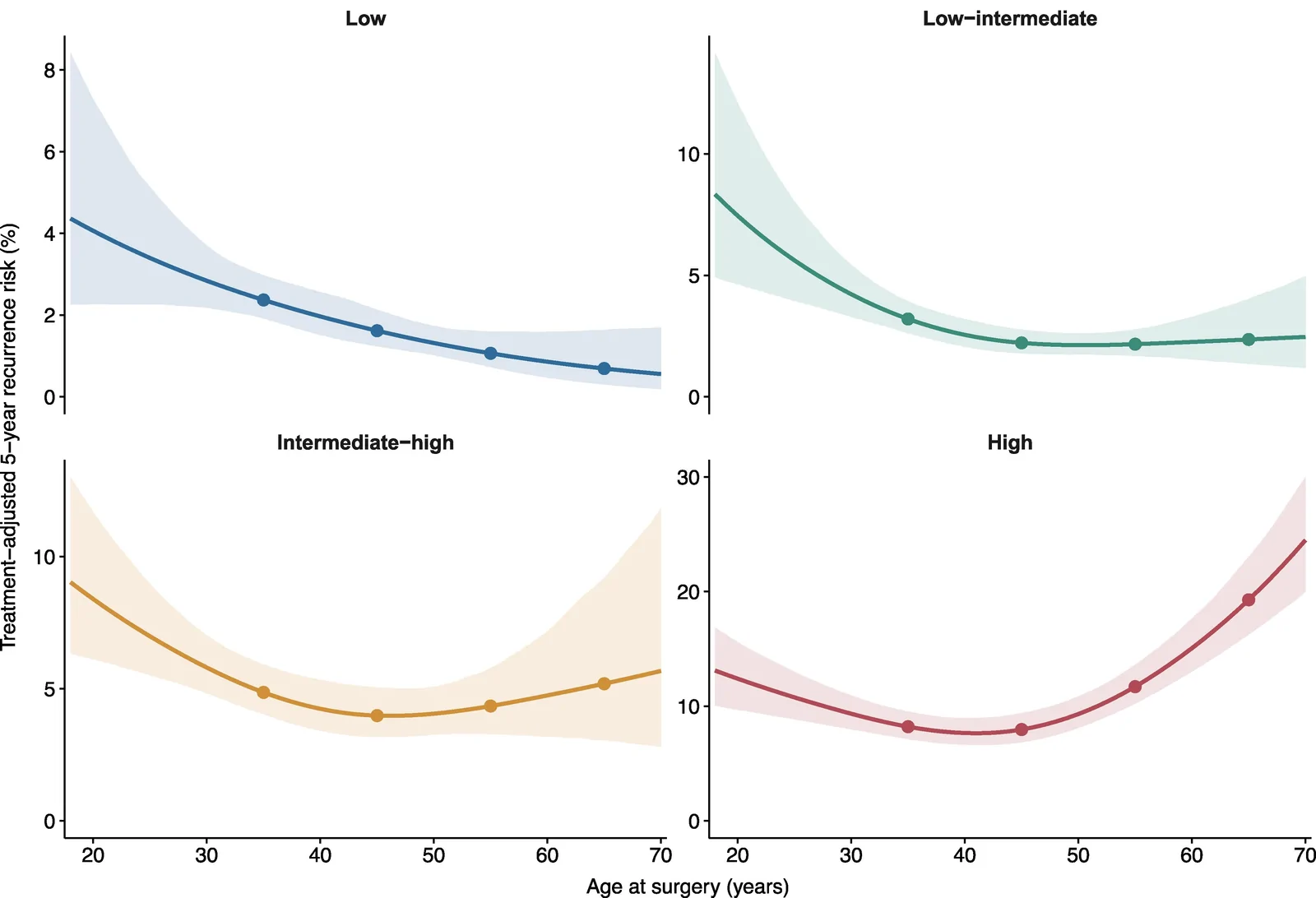
Treatment-adjusted age-specific 5-year structural recurrence risk according to ATA2025 recurrence risk stratification. Age was modeled with a restricted cubic spline with knots at 29, 43, and 58 years and interacted with ATA 2025 stratification. Curves were standardized over the observed joint distribution of sex, surgical period, surgery extent, central neck dissection, and postoperative treatment. Shaded bands indicate 95% confidence intervals from 2000 coefficient simulations; points mark the prespecified ages of 35, 45, 55, and 65 years. Abbreviations: ATA, American Thyroid Association; CI, confidence interval.

The distribution of ATA 2025 stratum changed across surgical periods: the proportion classified as high risk was 29.7% in 2000-2009, 18.5% in 2010-2014 and 18.6% in 2015-2018, whereas the low-intermediate-risk proportion increased from 15.8% to 31.0% and 38.5% (Table S2) (14). Within the high-risk group, the treatment-adjusted risk difference between ages 65 and 35 years was 13.45 percentage points (95% CI 7.44-20.60) in 2000-2009, 7.73 (3.69-13.20) in 2010-2014 and 16.34 (10.09-24.11) in 2015-2018. The age-by-ATA association did not differ detectably across periods (three-way interaction *P* = .585; Fig. S4 and Table S5) (14).

The direction of the age association was preserved across alternative spline specifications, linear and categorical age models, treatment adjustment, follow-up restrictions, restriction to patients receiving thyroid-stimulating hormone suppression alone, exclusion of older patients and substitution of last follow-up time for selected censored observations. Across these analyses, the age-65-vs-age-35 risk difference remained negative in the low-risk category and positive in the high-risk group (all global interaction *P* < .001; Fig. S5 and Table S6) (14).

### Temporal validation, decision analysis, and reclassification

The temporal derivation cohort included 10 357 patients with 606 recurrences, and the validation cohort included 4040 patients with 152 recurrences. In validation, the ATA-based and age-interaction models had similar discrimination: Harrell C-indices were 0.745 (95% CI 0.701-0.797) and 0.745 (0.701-0.798), and 5-year time-dependent areas under the curve were 0.743 (0.697-0.789) and 0.747 (0.700-0.792), respectively. The corresponding Brier scores were 0.043 (0.037-0.050) and 0.042 (0.036-0.049), and calibration slopes were 1.299 (1.089-1.561) and 1.287 (1.079-1.553). Mean predicted 5-year risks were 4.40% (4.31-4.50) and 4.50% (4.40-4.62), compared with an observed risk of 4.74% (3.95-5.50) (Fig. 3A, Table 3, and Table S7A) (14).

**Figure 3:**
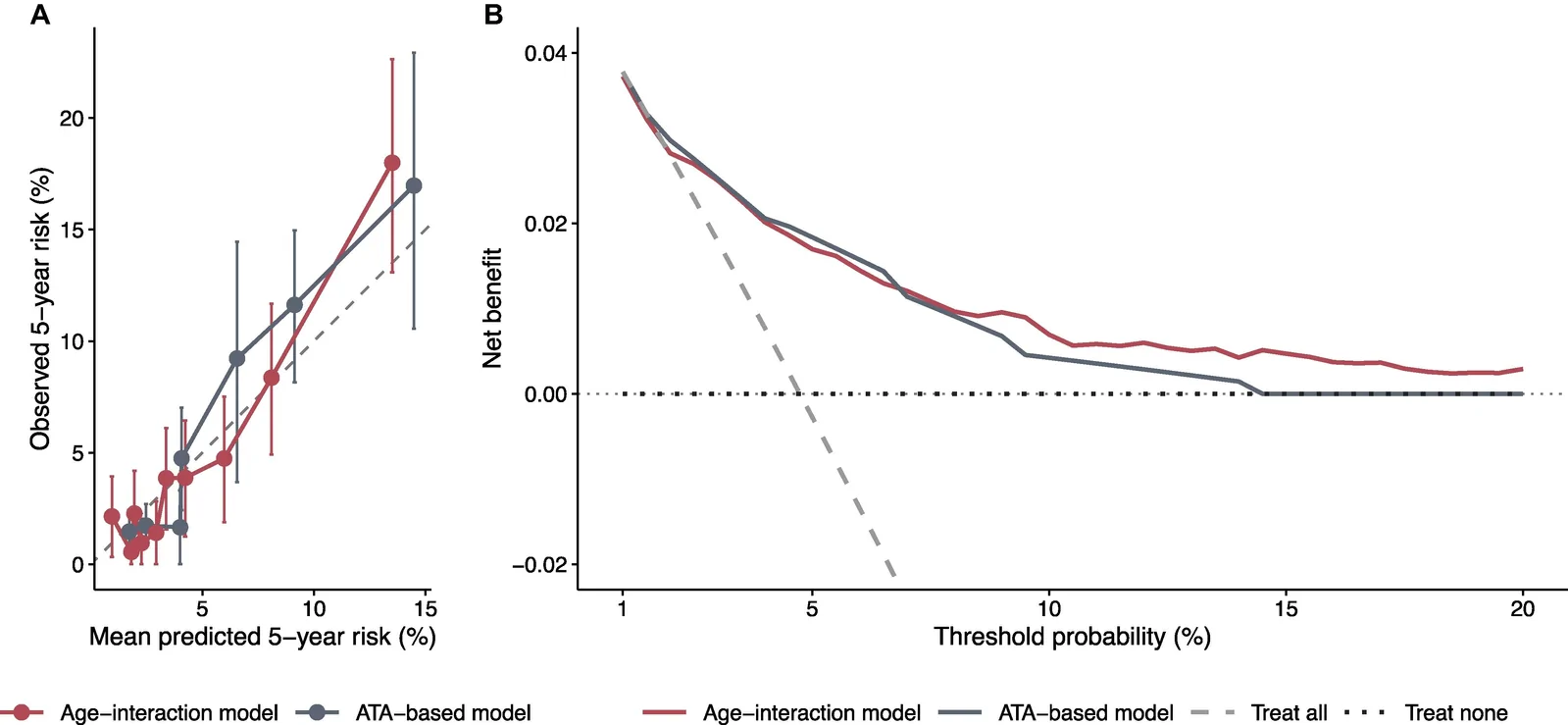
Temporal validation of the ATA-based and age-interaction models. Models were fitted in patients treated during 2000-2016 and evaluated in patients treated during 2017-2018. (A) Observed Kaplan–Meier 5-year risk plotted against mean predicted risk within quantile groups; error bars indicate 95% confidence interval, and the dashed line indicates perfect calibration. (B) Inverse-probability-of-censoring-weighted decision curves across threshold probabilities of 1% to 20%.

**Table 3:** Model performance in the temporal validation cohort

Metric	ATA-based model	Age-interaction model
Harrell C-index	0.745 (0.701-0.797)	0.745 (0.701-0.798)
5-Year time-dependent AUC	0.743 (0.697-0.789)	0.747 (0.700-0.792)
5-Year Brier score	0.043 (0.037-0.050)	0.042 (0.036-0.049)
Calibration slope	1.299 (1.089-1.561)	1.287 (1.079-1.553)
Mean predicted 5-year risk	4.40% (4.31-4.50)	4.50% (4.40-4.62)
Observed 5-year risk	4.74% (3.95-5.50)	4.74% (3.95-5.50)

Models were fitted in patients treated during 2000-2016 and evaluated in patients treated during 2017-2018. The ATA-based model included ATA 2025 stratification, sex, and surgical period. The age-interaction model additionally included spline-modeled age and age-by-ATA interaction terms. Discrimination was assessed using Harrell C-index and the inverse-probability-of-censoring-weighted 5-year time-dependent area under the receiver operating characteristic curve. Prediction error was assessed using the 5-year Brier score, and calibration was evaluated using the calibration slope and agreement between mean predicted and Kaplan–Meier-estimated risks. Values are estimates with 95% confidence intervals obtained from 300 bootstrap resamples of the validation cohort. ATA, American Thyroid Association; AUC, area under the receiver operating characteristic curve; CI, confidence interval.

Decision curves were closely aligned over most threshold probabilities (Fig. 3B). Compared with the ATA-based model, the age-interaction model had net-benefit differences of −0.0015 at a 2% threshold, −0.0014 at a 5% threshold and 0.0027 at a 10% threshold; the latter corresponded to 2.44 additional net interventions avoided per 100 patients (Table S7B) (14).

In the apparent full-cohort analysis, the age-interaction model assigned 3779 patients (26.2%) to a lower risk group, left 9144 (63.5%) unchanged and assigned 1474 (10.2%) to a higher risk group. In temporal validation, 627 patients (15.5%) moved lower, 2788 (69.0%) remained unchanged and 625 (15.5%) moved higher. Their observed 5-year risks were 2.29% (95% CI 0.67-3.87), 5.18% (4.11-6.23), and 5.10% (2.85-7.30), respectively. Within the original high-risk group, observed risk was 11.88% (8.69-14.96) among 632 patients who remained in the same group and 21.30% (11.21-30.24) among 106 patients who moved higher (Fig. S6 and Tables S8A and S8B) (14).

## Discussion

In this large retrospective cohort of patients with PTC, ATA2025 recurrence risk stratification separated 5-year structural recurrence risk after initial surgery. However, the absolute recurrence risk represented by each stratum varied by age. Incorporating continuous age and its interaction with ATA2025 stratum showed that predicted recurrence risk declined with older age in the low-risk stratum, remained low in the low-intermediate-risk stratum, changed little in the intermediate-high-risk stratum, and increased substantially at older ages in the high-risk stratum. The age-by-ATA interaction persisted after adjustment for surgery and postoperative treatment, across surgical periods, and in sensitivity analyses. Its incremental predictive value was limited, however: temporal-validation discrimination was essentially unchanged, and the decision curves differed only modestly. These findings position age as a potential modifier of ATA 2025 risk, particularly in high-risk disease, rather than as a replacement for the guideline system or a stand-alone basis for treatment decisions (4).

The high-risk pattern is consistent with earlier studies conducted under the 2015 ATA framework. Shah and Boucai reported poorer response to therapy and higher disease-specific mortality among older patients with ATA high-risk disease (15). In a multicenter cohort, Trimboli et al found that age 55 years or older was associated with relapse primarily in the high-risk group (7), and van Velsen et al subsequently showed adverse associations between continuous age and recurrence, treatment response, and disease-specific mortality in high-risk differentiated thyroid cancer (10). Maino et al also observed that the prognostic contribution of older age in low- and intermediate-risk disease was concentrated among patients with intermediate-risk features and persistent structural disease (11). Building on our earlier analysis of a partly overlapping cohort, which identified a U-shaped association between age and recurrence in papillary thyroid carcinoma (12), the present study shows that this overall pattern is not uniform across ATA 2025 stratifications. Modeling age continuously within the 4-category system revealed little change across age in the 2 intermediate groups and a pronounced positive age gradient in the high-risk group.

The results also extend work linking age-based mortality staging with recurrence-risk classification. Ghaznavi et al showed that ATA risk stratifications refined disease-specific survival estimates within age-defined AJCC stage groups (16). The present analysis addresses the complementary question: age refined absolute structural recurrence risk within ATA-defined groups. Together, these observations indicate that age and clinicopathological recurrence risk contains related but nonredundant prognostic information. This distinction matters because mortality staging, initial recurrence-risk estimation, and response-to-therapy assessment serve different clinical purposes and should not be used interchangeably (4, 17).

The absolute difference within the high-risk group was clinically substantial. At age 65 years, the treatment-adjusted 5-year structural recurrence risk was 19.28%, compared with 8.21% at age 35 years, an 11.07-percentage-point difference. Age may capture variation in tumor phenotype that is not fully represented by conventional pathological features. Recent molecular data in high-risk differentiated thyroid cancer suggest that late-hit alterations involving the TERT promoter, TP53, or PIK3CA identify patients with greater structural recurrence risk (18). Such alterations are also more common in older patients, raising the possibility that age partly marks unmeasured molecular risk. The present data cannot separate biological aging from molecular profile, comorbidity, treatment selection, or other age-related factors. For current practice, the most direct use of the observed interaction is therefore in risk communication and in defining populations for closer prospective study, not in escalating surgery or radioactive iodine solely because of age.

The lower-risk findings reinforce that age is not a uniform adverse factor for structural recurrence. The low-risk group had a small absolute event rate throughout the age range, with lower modeled risk at older ages, whereas the low-intermediate- and intermediate-high-risk estimates were comparatively stable. This pattern may reflect differences in disease biology, case selection, or the distribution of unmeasured features within each risk stratification. It should not be interpreted as evidence that surveillance can be reduced for an individual older patient. ATA risk assessment is intended to support risk-adapted care and to be updated with postoperative response data over time (4, 17). Age-specific estimates are best considered one component of that process.

Evaluation in the temporal validation cohort clarified the practical contribution of age. The ATA-based and age-interaction models had the same Harrell C-index of 0.745; the 5-year area under the curve increased only from 0.743 to 0.747, and the Brier score changed from 0.043 to 0.042. Mean predicted risks were close to the observed 5-year risk for both models. Decision curves were also similar: the age-interaction model had slightly lower net benefit at 2% and 5% thresholds and a small advantage at 10%, equivalent to 2.44 additional net interventions avoided per 100 patients. These results distinguish statistical interaction from incremental clinical utility. Prediction models require adequate calibration and demonstrable decision value before clinical adoption (19, 20). Age changed individual absolute-risk estimates, but it did not materially improve overall ranking or decision performance in this validation sample.

Reclassification showed the same conditional value. In temporal validation, 31.0% of patients moved to a different prespecified risk category, yet observed 5-year risks were similar for the overall moved-higher and unchanged groups. Within the original high-risk category, the signal was more distinct: observed risk was 21.30% among patients moved higher and 11.88% among those who remained in the same category. The confidence interval for the moved-higher group was wide, and this subgroup contained 106 patients. Reclassification by age may therefore be most informative within an already high-risk population, but the proposed thresholds require external validation before they are linked to surveillance or treatment pathways.

Treatment patterns provide important context for these estimates. ATA 2025 stratification were assigned retrospectively and did not guide care delivered from 2000 through 2018. Accordingly, 35.9% of patients later classified as high risk had undergone lobectomy, 21.5% had received radioactive iodine, and 10 high-risk patients had received external-beam radiotherapy. Adjustment for surgery extent, central neck dissection, radioactive iodine, and external-beam radiotherapy produced nearly identical age-specific risk differences. The high-risk age gradient was also present in each surgical period, with no detectable age-by-ATA-by-period interaction. These analyses show that the principal association was not explained by the measured treatment variables or by a single treatment era. They do not estimate causal treatment effects, because treatment allocation was nonrandom and reflected the standards, information, and patient factors available at the time.

Several design features strengthen the analysis. The cohort was drawn from 18 785 evaluated patients and restricted to 14 397 with the complete pathological variables needed for ATA 2025 classification and complete follow-up; missing pathological variables were not imputed or assumed to be absent. Algorithm-generated classifications were reviewed against source records by 2 thyroid surgeons. Recurrence was defined structurally and confirmed by cytology, histopathology, or appropriate imaging, while scheduled surveillance included physical examination and neck ultrasonography every 6 months for the first 5 years and annually thereafter. The analysis treated age as a continuous, potentially nonlinear variable, reported standardized absolute risks, separated radioactive iodine from external-beam radiotherapy, examined surgical periods, and included temporal validation and multiple sensitivity analyses.

The study also has limitations. Although sex was included as an adjustment covariate in the nested Cox prediction models, men constituted only 24.5% of the cohort, and sex-specific effect modification was not evaluated. The age-by-ATA estimates should therefore not be assumed to be identical in men, and external validation in cohorts with greater male representation is warranted. It was retrospective and conducted at a single tertiary cancer center, and complete-case inclusion may limit transportability. Pathological reporting and treatment practice evolved over the 19-year accrual period, leaving potential residual misclassification and confounding despite source review, period analysis, and treatment adjustment. The cohort included only papillary thyroid carcinoma, and the findings should not be extrapolated to follicular or oncocytic carcinoma. Thyroglobulin and thyroglobulin antibody were not recurrence criteria; the endpoint therefore represents clinically identifiable structural disease rather than biochemical persistence or recurrence. A formal head-to-head comparison with dynamic risk stratification was not performed because the historical cohort lacked standardized longitudinal thyroglobulin, antithyroglobulin antibody, and imaging data required for consistent retrospective assignment of response-to-therapy categories. Death without documented recurrence was censored, so the analysis did not estimate recurrence in a competing-risk framework. Molecular data, body mass index, and interactions with other candidate modifiers were not evaluated. Events were sparse among older low-risk patients, and temporal validation within the same institution does not establish geographic or health-system transportability.

In conclusion, age modified the absolute 5-year structural recurrence risk represented by ATA 2025 stratifications, with the clearest separation in high-risk disease. The interaction was stable after measured treatment adjustment and across surgical periods, but it produced little overall gain in discrimination or net benefit. Age should therefore be evaluated as a targeted refinement of high-risk recurrence assessment, alongside molecular and dynamic response information, in external multicenter cohorts before it is used to change treatment or surveillance recommendations.

## Data Availability

Deidentiﬁed patient data will be available upon reasonable request to the corresponding author (via email) after institutional approval and with a signed data access agreement, with no time limits after publication, and with the permission of Cancer Hospital, Chinese Academy of Medical Sciences.
